# Increased expression of YTHDF1 and HNRNPA2B1 as potent biomarkers for melanoma: a systematic analysis

**DOI:** 10.1186/s12935-020-01309-5

**Published:** 2020-06-15

**Authors:** Tengda Li, Mingli Gu, Anmei Deng, Cheng Qian

**Affiliations:** 1 State Key Laboratory of Experimental Hematology, National Clinical Research Center for Blood Diseases, Institute of Hematology & Blood Diseases Hospital, Chinese Academy of Medical Sciences & Peking Union Medical College, Tianjin, 300020 China; 2 Department of Laboratory Diagnosis, Changhai Hospital, The Second Military Medical University, Shanghai, 200433 China; 3 Changhai Hospital, The Second Military Medical University, Shanghai, 200433 China; 4 Department of Laboratory Medicine, Shanghai Municipal Hospital of Traditional Chinese Medicine, Shanghai University of Traditional Chinese Medicine, Shanghai, 200071 China

**Keywords:** Melanoma, YTHDF1, HNRNPA2B1, m^6^A, Systematic analysis, p53

## Abstract

**Background:**

The incidence and mortality of melanoma is increasing around the world. To deeply explain the mechanism insight into it, we conducted a systematic analysis to examine the levels of regulatory genes of the common RNA epigenetic modification-N6-methyladenosine (m^6^A) in patients with melanoma compared by the healthy.

**Methods:**

We analyzed the expression of m^6^A Eraser, Writer, and Reader genes based on publicly available datasets on Oncomine and validated the results with a gene expression omnibus dataset. Hub genes were identified with Cytohubba and the frequency of copy number alterations was analyzed with the cBioPortal tool.

**Results:**

The results revealed the up-regulation of YTHDF1 and HNRNPA2B1 in melanoma. Combining the two genes improved the efficacy in diagnosing melanoma by about 10% compared to each gene alone. Hub genes identified with four analysis methods were compared and the overlapping genes were selected. These genes were enriched in several gene ontology terms. Genes related to p53-signaling consisted of CDK2, CDK1, RRM2, CCNB1, and CHEK1. All five genes were positively correlated with either YTHDF1 or HNRNPA2B1, suggesting that both genes may affect m^6^A modification by the five genes, further up-regulating their expression and facilitate their roles in inhibiting p53 to suppress tumorigenesis. We also observed major mutations in YTHDF1 and HNRNPA2B1 that led to their amplification in melanoma. Significant differences were observed in the clinical characteristics of patients with altered and unaltered m^6^A regulatory genes such as tumor stage and treatment response.

**Conclusions:**

We, for the first time, identified a combination of m^6^A regulatory genes to diagnose melanoma. We also analyzed m^6^A-related genes more comprehensively based on systematic complete data. We found that YTHDF1 and HNRNPA2B1 were altered in melanoma and might influence the development of the disease through signaling pathways such as p53.

## Background

Melanoma is one of the fastest developing malignancies with strong aggressive ability, however no proper curative treatments exist at present [1, 2]. It is a type of epithelial malignant tumor originating from melanocytes with obviously increasing incidence and mortality around the world [1]. Based on insight into the molecular mechanism underlying the disease, serum lactate dehydrogenase is considered as a biomarker to predict the development of melanoma [3].

With the advancement of sequencing technology, related mutations such as B-Raf proto-oncogene (BRAF) and signaling pathways such as the mitogen-activated protein kinase (MAPK) pathway have been found. These discoveries have led to the emergence of targeted drug treatment for melanoma such as inhibitors of BRAF, mitogen-activated protein kinase kinase 7 (MAP2K7, also known as MEK), and mitogen-activated protein kinase 1 (MAPK1, also known as ERK) [2, 4, 5]. However, the actual pathological mechanism in melanoma remains unknown.

N6-methyladenosine (m^6^A) is a type of RNA epigenetic modification [6]. Originally reported only in mRNA, experts have found m^6^A in other types of RNA and involved in various bioprocesses with the development of high-throughput sequencing technology [7]. Similar to DNA methylation modification, m^6^A is also regulated by methyltransferase and demethylase [7, 8]. Previous reports showed that m^6^A methylation mainly interacted with three classes of proteins: firstly, m^6^A methyltransferase whose coding genes were called Writers and included methyltransferase-like 3 (METTL3), methyltransferase-like 14 (METTL14), and WT1 associated protein (WTAP); secondly, m^6^A demethylase whose coding genes were called Erasers and included alpha-ketoglutarate dependent dioxygenase (FTO) and alkB homolog 5 (ALKBH5); thirdly, proteins that could attach to the m^6^A methylation site in RNA and further played a specific role in physio-pathological processes with coding genes called Readers, which included YTH N6-methyladenosine RNA-binding protein 1 (YTHDF1), YTH N6-methyladenosine RNA-binding protein 2 (YTHDF2), E74-like ETS transcription factor 3 (ELF3), heterogeneous nuclear ribonucleoprotein C (HNRNPC), and heterogeneous nuclear ribonucleoprotein A2/B1 (HNRNPA2B1) [6, 9]. Recent studies have shown that m^6^A methylation is closely related to tumorigenesis and tumor development [10]. Vu et al. [11] reported that CD34+ hematopoietic stem cells with lower expression of METTL3 had higher levels of phosphorylated protein kinase B and that METTL3 promoted normal stem cells into acute myeloid leukemia cells. Zhou et al. [12] reported increased expression of FTO in tissue lesions of cervical squamous cell carcinoma (CSCC) and observed that patients with high FTO expression exhibited chemotherapy tolerance. However, few studies have investigated the role of m^6^A modification in the development or pathological process of melanoma.

**Fig. 1:**
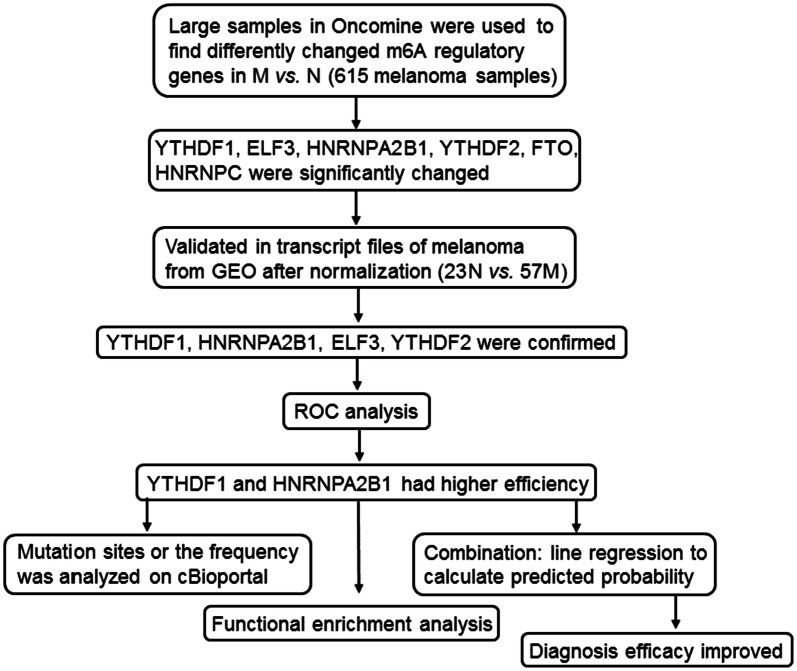
Flow chart of study. M, melanoma; N, normal; ROC, receiver operating characteristic curve

In this research, our goal was to examine the expression of m^6^A regulatory genes in melanoma based on open biological data and to identify the related pathways. Firstly, we used the online Oncomine tool to determine whether the m^6^A-related genes presented above were altered in melanoma. We then extracted the RNA-seq data from the gene expression omnibus (GEO) website (http://www.ncbi.nlm.nih.gov/geo) to validate the results from Oncomine and investigate the altered genes. Next, we used statistical methods to evaluate the hub genes involved in RNA processing and calculate the correlation between the hub genes and the selected target genes. Finally, we analyzed all the critical genes and identified the signaling pathways and mutation hot spots for m^6^A regulatory genes to uncover new therapy targets and determine the m^6^A-related biological mechanism in melanoma. A summary of this study is shown in Fig. 1.

## Methods

### Ethics statement

All clinical data, copy number alterations (CNAs), mutations, RNA-seq data, and other data were abstracted from Oncomine, cBioPortal (http://cbioportal.org), or the GEO platform, which are open to the public. All analyses were performed based on the data of previously published studies. Therefore, no ethical approval or patient consent was required for this study.

### Overview of the expression of 10 genes in Oncomine

**Fig. 2:**
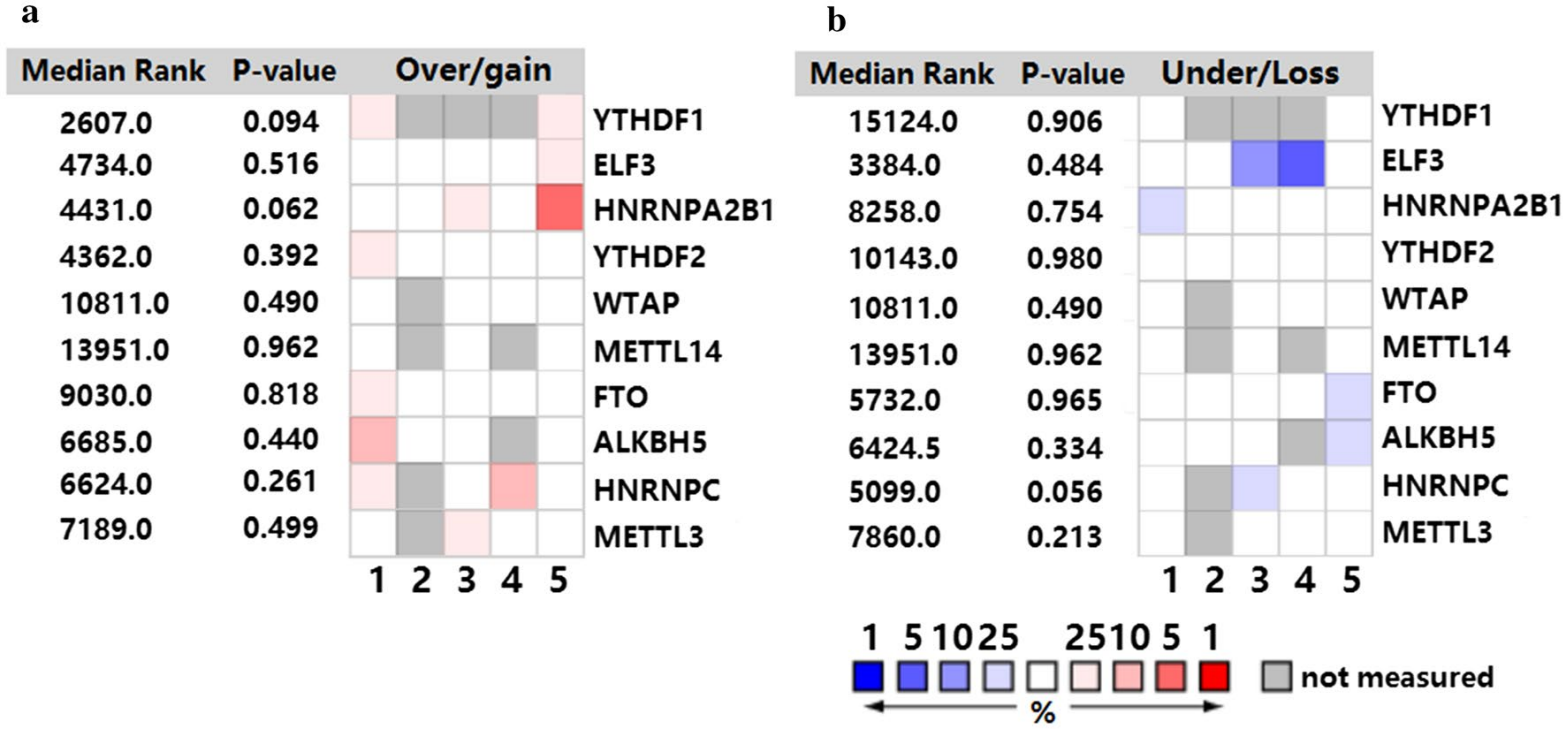
Overview of the expression of 10 m^6^A-related genes in melanoma from Oncomine. Comparison of over-expression (**a**) and under-expression (**b**) of different genes among the selected studies. 1 represents the study by Critchley-Thorne et al., 2007; 2 represents Haqq et al., 2005; 3 represents Riker et al., 2008; 4 represents Talantov et al., 2005; 5 represents TCGA, 2013; the rank for a gene is the median rank for the gene across each of the analysis methods and the *P*-value is for the median-ranked analysis

The Oncomine (https://www.oncomine.org) tool was used to determine the expression of m^6^A regulators in melanoma, five studies (Haqq et al. 2005; Talantov et al. 2005; TCGA 2003; Riker et al. 2008; Critchley et al. 2006) that had mRNA or DNA expression documents were selected. There were total 615 patients with melanoma included in this research, and their information was presented in Additional file 1: Table S1. We used “Differential analysis” online tool in Oncomine portal to systematically compare the DNA or transcript levels of the normal and patients with melanoma. The whole data in the five studies above were corrected and then median ranked analysis was performed, results were shown in Fig. 2.

### Validation of the expression of all genes by RNA-seq data from GEO

We obtained the RNA-seq data from GEO (23 normal *vs* 57 patients with melanoma). Patient information is shown in Additional file 1: Table S2. R package affyPLM and RColorBrewer were used to do the quality control. Robust Multi-Array Average method was used in data pre-processing, then the files from the normal and patients’ samples were merged. Package impute in R was used to normalize and correct the files and package limma was to determine the different expression files. Heat map and volcano graphs were drew by gplots package in R software. The expression files of target genes were abstracted to do further analysis.

### Functional enrichment analysis of the normalized RNA expression files from GEO

Gene ontology (GO) term and Kyoto Encyclopedia of Genes and Genomes analysis were performed by ClueGO [13, 14]. Biological Process, Cellular Component, Molecular Function, Immune System Process ontologies and KEGG pathways were selected. GO tree interval was from 3 to 8, the minimal number of genes in GO term or pathway was 3. GO term/pathway network connectivity (Kappa Score) was 0.4, the statistical option was Enrichment/Depletion (Two-sided hypergeometric test) and pV correction was Bonferroni step down. The String tool (https://string-db.org/) was used to draw the general network. Cytoscape (CytoHubba) was used to predict and explore the critical genes, nodes’ score was calculated in this software and the scores of maximal clique centrality (MCC), maximum neighborhood component (MNC), edge percolated component (EPC), and degree methods of each genes were obtained to determine the key genes in this study.

### Analysis of mutation diagram for each gene with cBioPortal

We used cBioPortal to analyze the frequency of mutations and CNAs in patients with melanoma. A total of 653 samples were included in this research. Detailed information for the patients is shown in Table 1. Oncoprint tool in cBioPortal was used to scan mutation frequency of each gene in every sample, samples with mutation were presented in Fig. 5. There were three studies (Snyder, MEJM; TCGA; Van Allen, Science) included in this research. Cancer Type tool was used to show the mutation types of every gene or total genes in each study. Mutations tool in cBioPortal was used to showed the mutation sites for each gene in melanoma, and the protein post-translational modification (PTM) site plugin in mutations tool was used to predict the occurrence of phosphorylation, acetylation, ubiquitination, methylation, and O-linked glycosylation, primarily between 0 and 370aa.

**Table 1:** Clinical information for altered and unaltered groups on cBioPortal

Clinical attribute	Statistical test	*P*-value	*Q*-value
Somatic status	Chi squared test	1.43 * 10^−5^	9.68 * 10^−4^
Biopsy time	Chi squared test	2.20 * 10^−5^	9.68 * 10^−4^
Cohort	Chi squared test	2.86 * 10^−5^	9.68 * 10^−4^
Tumor stage	Chi squared test	3.34 * 10^−5^	9.68 * 10^−4^
HLA_DPA2	Chi squared test	1.23 * 10^−4^	2.84 * 10^−4^
Tumor site	Chi squared test	3.12 * 10^−4^	4.71 * 10^−4^
Mutation count	Kruskal–Wallis test	3.76 * 10^−4^	4.71 * 10^−4^
Disease free status	Chi squared test	4.02 * 10^−4^	4.71 * 10^−4^
Cancer type detailed	Chi squared test	4.11 * 10^−4^	4.71 * 10^−4^
Oncotree code	Chi squared test	4.11 * 10^−4^	4.71 * 10^−4^
HLA_DPB2	Chi squared test	4.70 * 10^−4^	4.71 * 10^−4^
Durable clinical benefit	Chi squared test	4.87 * 10^−4^	4.71 * 10^−4^
RAF_RAS status	Chi squared test	6.57 * 10^−4^	5.49 * 10^−4^
HLA_DQA2	Chi squared test	6.68 * 10^−4^	5.49 * 10^−4^
HLA_DRB2	Chi squared test	7.09 * 10^−4^	5.49 * 10^−4^
Neo-antigen load	Kruskal–Wallis test	1.24 * 10^−3^	9.02 * 10^−4^
Serum lactate dehydrogenase	Kruskal–Wallis test	1.75 * 10^−3^	0.012
Dosage	Chi squared test	2.76 * 10^−3^	0.0178
Mutation load	Kruskal–Wallis test	3.62 * 10^−3^	0.021
Treatment response	Chi squared test	3.82 * 10^−3^	0.0211

### Statistics

For the comparison of two different groups, the *t* test or Mann-Whitney test was used according to the data distribution. The Chi squared test was performed to compare the discrete data and the Kruskal–Wallis test was used to compare the expression of more than two groups that did not conform to the conditions of the parametric test. The relationship between two continuous variables was evaluated with the Pearson correlation coefficient. Receiver operating characteristic (ROC) curves and scatter diagrams were drawn using GraphPad Prism 6.0. IBM SPSS Statistics version 21.0 was used to calculate all statistical parameters. The significance level was *P *< 0.05.

## Results

### Systematic comparison of m^6^A regulatory genes in melanoma and normal populations

We used the Oncomine database to compare the expression of m^6^A-related genes in patients with melanoma and healthy individuals reported in five studies. The inclusion criteria were as follows: patients involved in the studies had a definite diagnosis or histopathological diagnosis of melanoma; the samples were from patients confirmed to have clinical data; the data were collected from tissue samples not cell lines; gene expression profiles were available. We excluded the datasets if : they were without clinical information; the samples’ type was cell line or primary cell culture; their gene expression files were not provided on the Oncomine portal; they did not have samples of both patients with melanoma and the healthy. Samples were included in this study regard less of the age, race or sex. As shown in Fig. 2, YTHDF1 and HNRNPA2B1 were significantly over-expressed in patients with melanoma while ELF3 was down-regulated. The detailed *P*-values are shown in Table 2. Other genes such as HNRNPC were also altered to some degree. We further validated our findings using normalized RNA-seq data from GEO.

**Table 2:** *P*-values of different genes in five studies selected in Oncomine

	Critchley	Haqq	Riker	Talantov	TCGA
YTHDF1	0.187	–	–	–	1.14 * 10^−7^
ELF3	0.258	0.516	0.887	0.999	3.86 * 10^−7^
HNRNPA2B1	0.814	1.197	0.062	0.246	2.64 * 10^−10^
YTHDF2	0.117	0.392	0.182	0.020	0.441
WTAP	0.794	–	0.185	0.400	1.000
METTL14	0.671	–	0.899	–	0.962
FTO	0.167	0.035	0.259	0.818	0.989
ALKBH5	0.092	0.668	0.211	–	0.995
HNRNPC	0.200	–	0.322	2.68 * 10^−5^	0.943
METTL3	0.349	–	0.074	0.650	0.944

The results are from comparisons of patients with melanoma and healthy individuals. Critchley, Critchley-Thorne, et al., Plos Med (2007); Haqq, Christopher Haqq, et al., PNAS(2005); Riker, Adam I Riker, et al., BMC Med Genomics (2008); Talantov, Dmitri Talantov, et al., Human cancer biology (2005); TCGA, the cancer genome atlas (2013)

### Validation of the expression of target genes

**Fig. 3:**
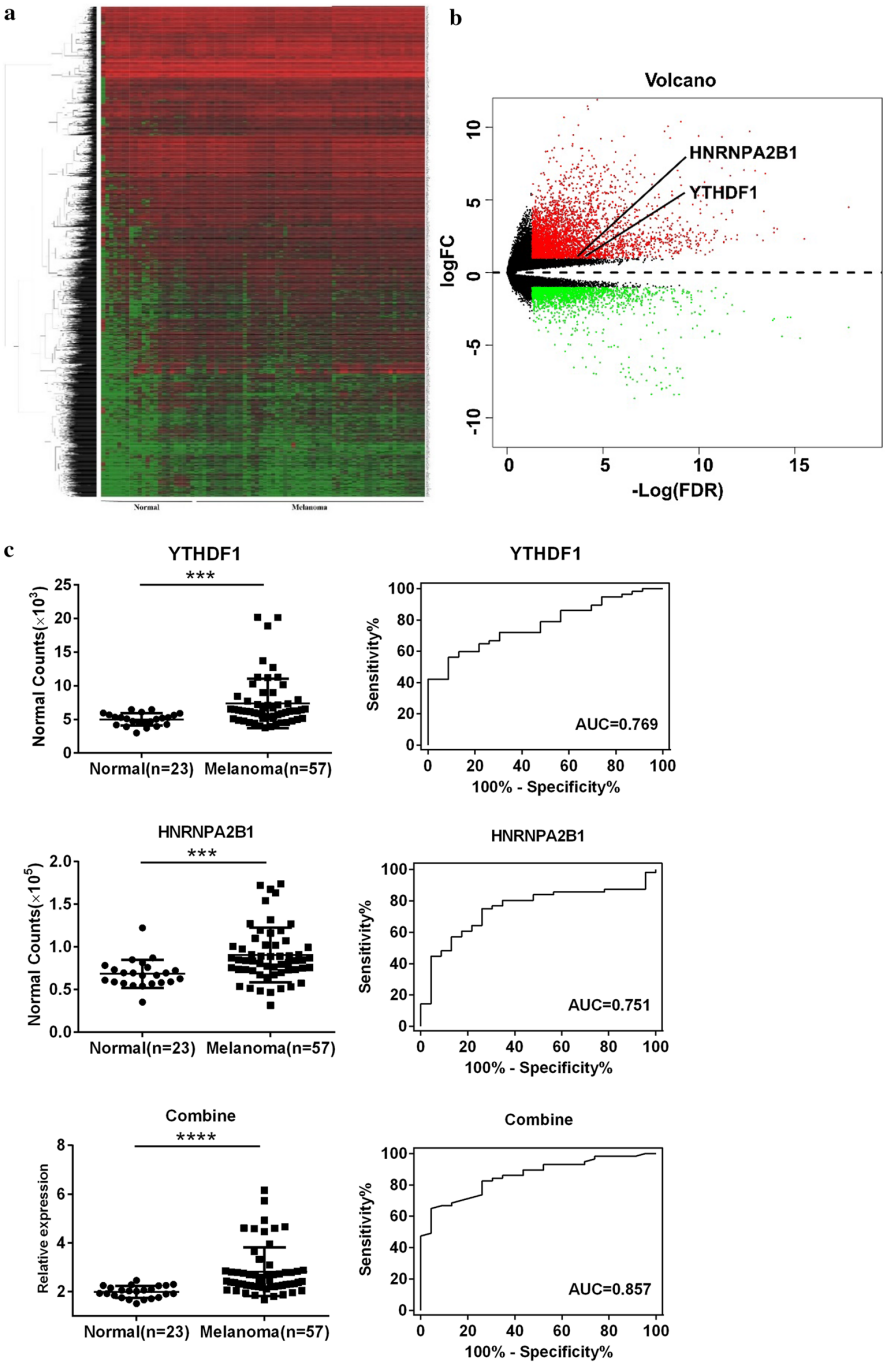
Expression of YTHDF1 and HNRNPA2B1 in the complete RNA-seq dataset from GEO. **a** Cluster heat map and **b** volcano plot of all genes in healthy individuals (n = 23) and patients with melanoma (n = 57); **b** red points represent up-regulated genes, green represents down-regulated genes. **c** Expression level and ROC curves of YTHDF1 and HNRNPA2B1 and their combination. FC, fold change; FDR, false discovery rate; AUC, area under curve; ROC, receiver operating characteristic curve. ***P < 0.001, ****P < 0.0001

Original RNA-seq files for 53 patients with melanoma and 27 healthy individuals were obtained from GEO. The gene expression for all participants is shown in a cluster heat map in Fig. 3a and the up-regulated (red) and down-regulated (green) genes are visually presented in volcano plots (Fig. 3b). We examined the 10 genes of interest in this dataset and found that only HNRNPA2B1, YTHDF1, ELF3, and YTHDF2 were significantly altered in patients with melanoma (Table 3, Fig. 3c, Additional file 2: Figure S1). Integrating the results from GEO and Oncomine, the expression of HNRNPA2B1, YTHDF1, and ELF3 showed the same patterns and were thus persuasive. We assessed the specificity and sensitivity of these three genes and observed that HNRNPA2B1 and YTHDF1 had higher ROC areas than YTHDF2, whereas ELF3 had a lower value (Table 3). Therefore, we selected HNRNPA2B1 and YTHDF1 for further research and combined the expression of the two genes to evaluate the combined specificity and sensitivity. The equation for the combination used in this study was X = logit (P) = ln (P/1 − P) = − 7.777 + 3.93*YTHDF1 + 1.024*HNRNPA2B1. Next, the predicted probability of melanoma was calculated with the following equation: predicted probability (P) = e^x^/(1 + e^x^) [15, 16]. The results indicated the efficiency of combining YTHDF1 and HNRNPA2B1 compared to the results for each gene individually, with an improvement of almost 10% (Fig. 3c, Table 3). The expression and ROC curves for ELF3, and YTHDF2 are presented in Additional file 2: Figure S1.

**Table 3:** The ROC curve-related parameters and results of Mann–Whitney test

	HNRNPA2B1	YTHDF1	ELF3	YTHDF2	Combined^a^
Area	0.755	0.769	0.682	0.711	0.857
95% CI	0.645–0.866	0.666–0.872	0.554–0.810	0.600–0.822	0.776–0.938
*P*-ROC	0.000380	0.000181	0.0113	0.00335	< 0.0001
*P*-MW	0.0004	0.0001	0.0107	0.0029	< 0.0001

*CI* confidence interval, *ROC* receiver operating characteristic curve, *MW* Mann–Whitney test. ^a^Combination of HNRNPA2B1 and YTHDF1, details are given in the methods section

### m^6^A-associated mechanism in melanoma

**Fig. 4:**
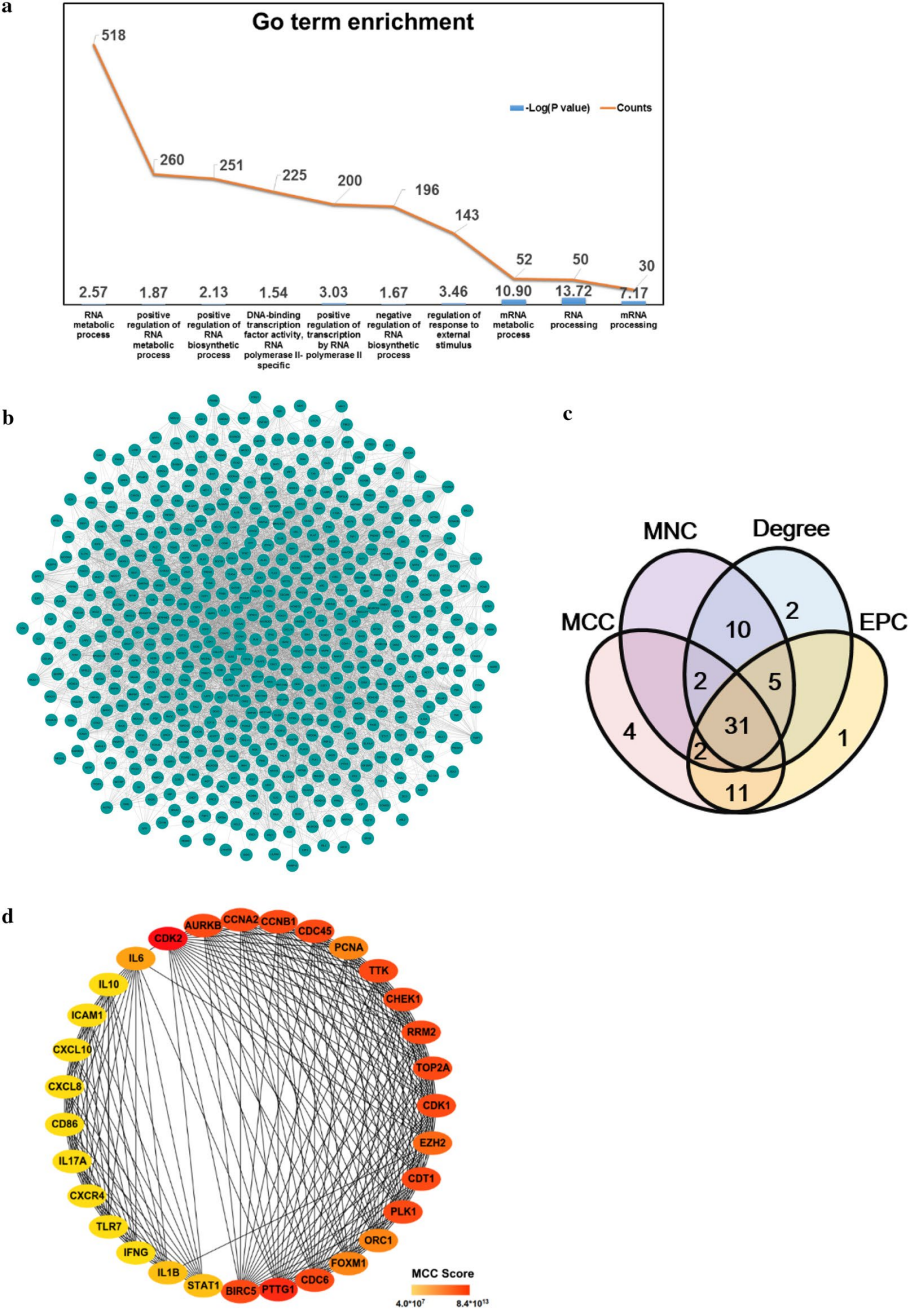

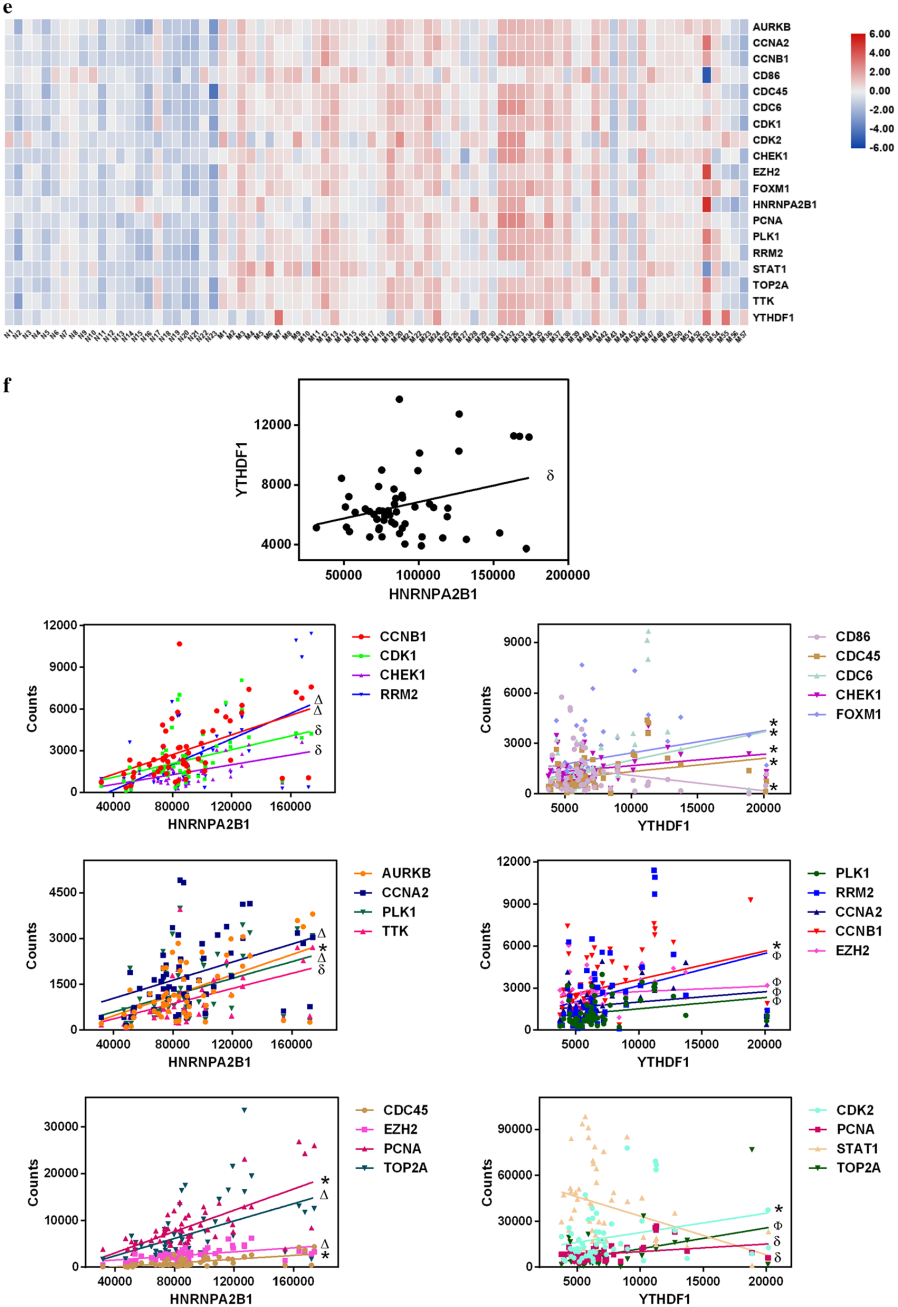

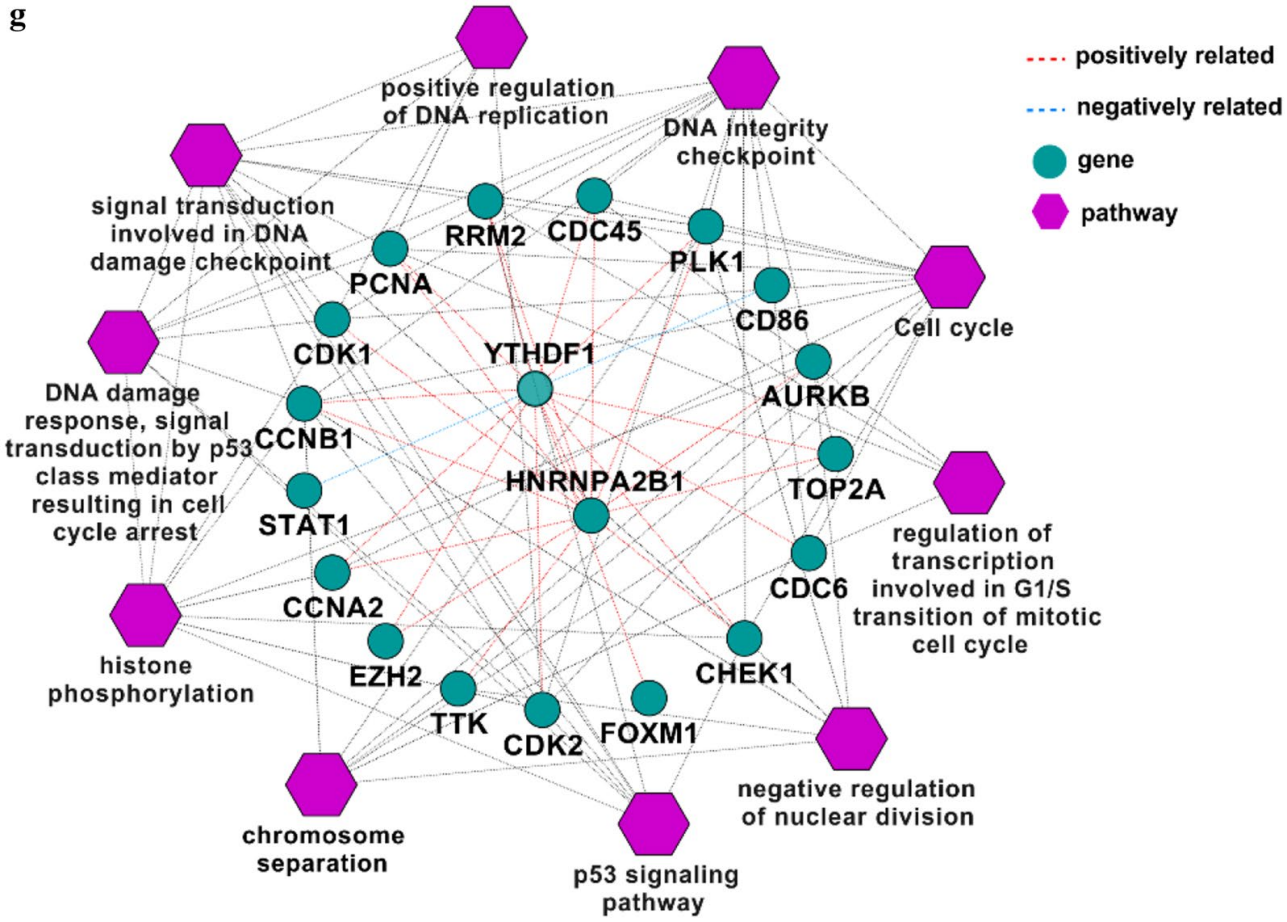
m^6^A-associated mechanism in melanoma. **a** Top 10 GO terms for RNA processing. **b** Network of all genes identified with the **a** String tool. **c** Venn diagram of genes identified with different analysis methods to determine the key genes in melanoma. **d** Network and rank scores of 31 hub genes found in the results from all four methods in (**c**). **e** Heat map of genes that had a significant relationship with YTHDF1 or HNRNPA2B1. **f** Scatter plots of expression of YTHDF1 and HNRNPA2B1 and all genes in (**e**) and their associations. **g** Pathway or GO terms of the critical genes in melanoma and their relationship with YTHDF1 or HNRNPA2B1. MCC, maximal clique centrality; MNC, maximum neighborhood component; EPC, edge percolated component. * *P *< 0.05, δ *P *< 0.01, Ф *P *< 0.001, Δ *P *< 0.0001

We extracted the GO terms for all genes using ClueGO and selected 611 genes involved in RNA processing or the epigenetic activation processes (Fig. 4a). After filtering the unconnected genes from the network using the String tool (Fig. 4b), we chose 586 nodes to determine the important genes using CytoHubba in Cytoscape. The top 50 genes according to the MCC, MNC, EPC and degree were extracted and a Venn diagram was created using Venny tool online. Thirty-one genes overlapped in the results from the four types of analysis (Fig. 4c). The network and MCC score heat nodes are illustrated in Fig. 4d, which shows that cyclin dependent kinase 2 (CDK2), CDK1, cyclin-B1 (CCNB1), and checkpoint kinase 1 (CHEK1) were altered. The detailed ranks for MCC and the other analysis methods are shown in Table 4. Further we performed the correlation analysis of all 31 genes and YTHDF1 or HNRNPA2B1 by calculating their Pearson correlation coefficients (Table 5). Only 17 genes were ether positively or negatively correlated with YTHDF1 or HNRNPA2B1 (*P *< 0.05) (Table 5).

**Table 4:** Evaluation of hub genes

Gene name	MCC	MNC	Degree	EPC
CDK2	8.37 * 10^13^	20	20	22.494
PTTG1	8.37 * 10^13^	19	19	22.596
CCNA2	8.37 * 10^13^	18	18	22.265
CDT1	8.37 * 10^13^	18	18	22.422
PLK1	8.37 * 10^13^	18	18	22.289
CCNB1	8.37 * 10^13^	18	18	22.443
CDC6	8.37 * 10^13^	18	18	22.361
CDK1	8.37 * 10^13^	18	18	22.219
CDC45	8.37 * 10^13^	18	18	22.28
AURKB	8.37 * 10^13^	18	18	22.373
CHEK1	8.37 * 10^13^	18	18	22.259
BIRC5	8.37 * 10^13^	18	18	22.234
TOP2A	8.37 * 10^13^	18	18	22.419
TTK	8.37 * 10^13^	18	18	22.399
RRM2	8.37 * 10^13^	18	18	22.558
EZH2	4.18 * 10^13^	19	19	22.315
ORC1	4.18 * 10^13^	17	17	22.183
FOXM1	4.18 * 10^13^	17	17	22.118
PCNA	4.18 * 10^13^	17	17	22.174
IL6	3.99 * 10^7^	14	14	17.89
IL1B	3.99 * 10^7^	12	12	16.57
STAT1	3.9 * 10^7^	12	12	16.531
IL10	3.99 * 10^7^	11	11	15.753
CXCL8	3.99 * 10^7^	11	11	15.608
CD86	3.99 * 10^7^	11	11	15.382
CXCL10	3.99 * 10^7^	11	11	15.847
IFNG	3.99 * 10^7^	11	11	15.407
ICAM1	3.99 * 10^7^	11	11	15.725
CXCR4	3.99 * 10^7^	11	11	15.385
IL17A	3.99 * 10^7^	11	11	15.498
TLR7	3.99 * 10^7^	11	11	15.623

*MCC* maximal clique centrality, *MNC* maximum neighborhood component, *EPC* edge percolated component

**Table 5:** Pearson correlation coefficient for HNRNPA2B1, YTHDF1, and hub genes. Co-gene, genes included in the correlation analysis

HNRNPA2B1			YTHDF1		
Co-Gene	*r*	*P*-value	Co-Gene	*r*	*P*-value
AURKB	0.3285	0.01260	HNRNPA2B1	0.4225	0.0011
BIRC5	0.07396	0.5846	AURKB	0.3340	0.0111
CCNA2	0.9032	< 0.0001	BIRC5	0.2893	0.0291
CCNB1	0.5159	< 0.0001	CCNA2	0.4642	0.0003
CD86	− 0.2100	0.1170	CCNB1	0.3171	0.0162
CDC45	0.2998	0.0235	CD86	− 0.2613	0.0496
CDC6	0.1966	0.1428	CDC45	0.2951	0.0258
CDK1	0.3468	0.0082	CDC6	0.3094	0.0192
CDK2	0.1078	0.4246	CDK1	0.2523	0.0583
CDT1	0.006366	0.9625	CDK2	0.2730	0.0399
CHEK1	0.3661	0.0051	CDT1	0.2054	0.1253
CXCL10	− 0.09646	0.4754	CHEK1	0.2744	0.0389
CXCL8	0.09121	0.4998	CXCL10	− 0.1303	0.3339
CXCR4	− 0.06854	0.6124	CXCL8	0.1499	0.2658
EZH2	0.9444	< 0.0001	CXCR4	− 0.1834	0.1722
FOXM1	0.2333	0.0807	EZH2	0.4331	0.0008
ICAM1	− 0.1330	0.3241	FOXM1	0.2759	0.0377
IFNG	− 0.1123	0.4057	ICAM1	− 0.1410	0.2956
IL10	− 0.08513	0.5289	IFNG	− 0.1651	0.2197
IL17A	− 0.03174	0.8147	IL10	− 0.1322	0.3268
IL1B	0.01783	0.8952	IL17A	0.1084	0.4223
IL6	0.02979	0.8259	IL1B	0.006448	0.962
ORC1	0.1146	0.3961	IL6	0.1744	0.1944
PCNA	0.2903	0.0285	ORC1	0.2086	0.1195
PLK1	0.8851	< 0.0001	PCNA	0.3449	0.0086
PTTG1	0.06679	0.6216	PLK1	0.4889	0.0001
RRM2	0.6920	< 0.0001	PTTG1	0.1802	0.1797
STAT1	− 0.1878	0.1618	RRM2	0.4621	0.0003
TLR7	− 0.1181	0.3816	STAT1	− 0.3399	0.0097
TOP2A	0.8651	< 0.0001	TLR7	− 0.193	0.1504
TTK	0.3573	0.0064	TOP2A	0.4532	0.0004
YTHDF1	0.4225	0.0011	TTK	0.2193	0.1012

We visualized the expression of each gene by creating a heat map (Fig. 4e) and found that all nodes were up-regulated. Figure 4f shows that the expression of HNRNPA2B1 and YTHDF1 was positively correlated (*P *< 0.05). CCNB1, CDK1, CHEK1, ribonucleotide reductase regulatory subunit M2 (RRM2), aurora kinase B (AURKB), cyclin A2 (CCNA2), serine/threonine-protein kinase (PLK1), dual specificity protein kinase (TTK), cell division control protein 45 (CDC45), enhancer of zeste homolog 2 (EZH2), proliferating cell nuclear antigen (PCNA), and DNA topoisomerase 2-alpha (TOP2A) were positively associated with the level of HNRNPA2B1 (*P *< 0.05) (Fig. 4f). In addition to CD86 and STAT1, which were negatively correlated with YTHDF1, other genes in Fig. 4f exhibited a positive relation with YTHDF1 (*P *< 0.05). The interactions of m^6^A-regulated genes measured with the cBioPortal tool are shown in Additional file 1: Table S3 and confirm the co-occurrence of YTHDF1 and HNRNPA2B1 (*P *= 0.001, *Q *= 0.013).

Next, we created a network map to visualize the connections between these genes and further explored the pathways behind them (Fig. 4g). YTHDF1 and HNRNPA2B1 interacted with targeted genes involved in bioprocesses such as the p53 signaling pathway and positive regulation of DNA replication, which might provide insight into the molecular mechanism underlying melanoma (Fig. 4g).

### Mutation and CNAs of m^6^A-related genes in patients with melanoma

**Fig. 5:**
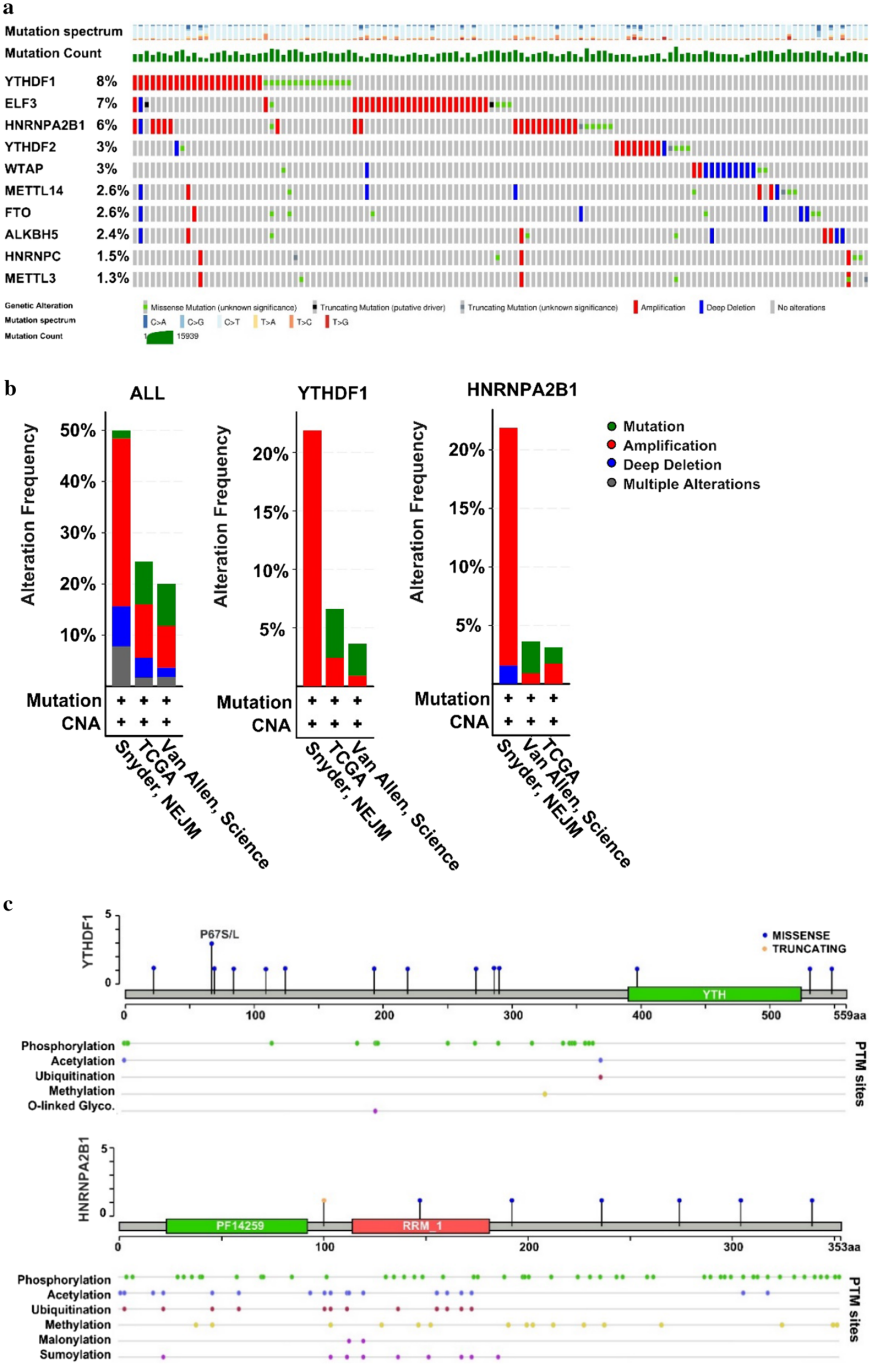
Copy number alteration (CNA) frequency for m^6^A-associated genes in melanoma. **a** Definition of the CNAs and their percentages for m^6^A regulators in melanoma. **b** The altered features in different studies. **c** Mutation diagram and PTM sites of YTHDF1 and HNRNPA2B1. PTM, protein post-translational modification; Glyco, glycosylation

We analyzed the data for melanoma patients with CNA and mutation profiles on cBioPortal. All mutated samples are shown in Fig. 5a. Integration of the mutation profiles with the clinical information of the patients revealed statistical differences between patients with alterations and those without alterations in terms of the tumor stage, durable clinical benefits, neo-antigen load, serum lactate dehydrogenase, and treatment response (Table 1). The mutation diagram shows the top three altered genes were YTHDF1 (8%), ELF3 (7%), and HNRNPA2B1 (6%), with a high frequency of amplification.

Three studies were included in our systematic analysis. The amplification of genes was dominant in Snyder’s study, while mutation and amplification were the major modifications in the other two studies (Fig. 5b). For YTHDF1 and HNRNPA2B1, the predominant alteration was amplification, followed by mutation (Fig. 5b). Other genes are shown in Additional file 2: Figure S2a.

Missense mutation was the most common alteration in YTHDF1, covering most of the gene (Fig. 5c). Missense and truncating mutations were observed in HNRNPA2B1. The PTM tool showed post-transcriptional modifications such as phosphorylation, acetylation, ubiquitination, methylation, malonylation, and sumoylation in the gene (Fig. 5c). The mutation frequency and PTM sites for YTHDF2, FTO, WTAP, ALKBH5, METTL14, HNRNPC, ELF3, and METTL3 are shown in Additional file 2: Figure S2b.

## Discussion

Melanoma is a tumor that consists of malignant melanocytes, which are pigment-producing cells of neuroectodermal origin that can be found throughout the body (including in the iris, rectum, and skin) [2]. With the advancement of science and technology coupled with increased recognition of the disease, experts’ interpretation of the pathogenesis of melanoma has changed from the previous explanation of simple molecular interactions to epigenetic regulation [17, 18]. As one of the most common chemical modifications in messenger RNA (mRNA), m^6^A has received substantial attention in epigenetics recently [10, 18]. Last year, Yang et al. [19] found that FTO, an m^6^A demethylase, could promote tumorigenesis and anti-PD-1 resistance in melanoma, highlighting the role of FTO in immunotherapy for the disease. In the same year, Jia et al. [18] observed a decrease in m^6^A levels in ocular melanoma samples, which could indicate poor prognosis and predict tumor progression. However, few studies based on systematic data have been conducted to explore m^6^A-related genes in melanoma and their general mechanisms. In this study, we summarized the expression of 10 Writer, Reader, and Eraser genes of m^6^A for the first time using Oncomine with more than 500 melanoma samples. We found that YTHDF1, ELF3, HNRNPA2B1, YTHDF2, FTO, and HNRNPC were significantly altered (Fig. 2, Table 2). By integrating the results from Oncomine and GEO, the up-regulation of HNRNPA2B1 and YTHDF1 was identified (Fig. 3, Table 3). We further examined whether the combination of YTHDF1 and HNRNPA2B1 had higher efficacy in diagnosing melanoma by using linear regression and found the efficacy was improved from about 0.760 to 0.857 (*P *< 0.0001). This combination provides the first optimized method to distinguish patients with and without melanoma based on m^6^A regulatory genes rather than common molecular biomarkers such as serum lactate dehydrogenase.

We also analyzed the GO terms for all genes. For the terms associated with RNA processing, 518 genes were enriched in RNA metabolic processes, with positive regulation of RNA metabolism as the secondary term. The genes for both processes were up-regulated, indicating the occurrence of dynamic and active RNA metabolic processes in melanoma (Fig. 4a). YTHDF1, an m^6^A Reader, was found to augment EIF3C translation by binding to m^6^A-modified EIF3C mRNA and to further promote tumorigenesis and metastasis in ovarian cancer [20]. Han et al. [21] demonstrated that targeting YTHDF1 with intestinal stem cells in established tumors could result in tumor shrinkage and longer survival. In our research, we found genes involved in the p53 signaling pathway such as CCNB1, CDK1, CHEK1, RRM2, and CDK2 were positively correlated with either YTHDF1 or HNRNPA2B1 (Fig. 4f). This suggested that YTHDF1 or HNRNPA2B1 may interact with the related genes above and further influence the p53 signaling pathway, resulting in the development of melanoma (Fig. 4g). CCNB1 silencing activates the p53 signaling pathway, further inhibiting the proliferation of cells and promoting cell senescence in pancreatic cancer [22]. CDK1/2 inhibitors activate p53 in tumors, especially the inhibitors of CDK2, which maintains the balance of S-phase regulatory proteins and thus coordinates subsequent p53-independent G2/M checkpoint activation [23, 24]. Hala et al. confirmed the existence of CHEK1/p53 association in human colorectal cancer in vivo and demonstrated that tumors lacking p53 had higher levels of CHEK1 [25]. The RRM1/RRM2B enzyme is capable of retaining activity in hypoxia and the replication pressure it induces is one of the factors proposed to promote selection stress that results in the loss of key ingredients for the DNA damage response, including p53 [26–29]. Therefore, YTHDF or HNRNPA2B1 might up-regulate CCNB1, CDK1, CHEK1, RRM2, or CDK2 (Fig. 4f), all of which could inhibit the role of p53 in suppressing tumors and promote the development of melanoma.

GO terms were found related to the activation of the cell cycle, DNA integrity checkpoints, histone phosphorylation, DNA damage response, signal transduction by p53 class mediation resulting in cell cycle arrest, regulation of transcription involved in the G1/S transition in the mitotic cell cycle, etc. (Fig. 4g). These findings suggested that the gene-interaction chain pathway might affect the proliferation as well as the normal physiological and metabolic processes of melanocytes, eventually resulting in the development of melanoma. All the genes in the identified pathways were also positively related to YTHDF1 or HNRNPA2B1 (Fig. 4f), suggesting the expression of both genes might influence m^6^A modifications in mRNA genes and potentially result in melanoma heterogeneity at the cellular level. As shown in Fig. 4f–g, STAT1 and CD86 were negatively regulated by YTHDF1. Previous studies demonstrated that the loss of STAT1 activation or expression was found in malignant cells derived from various tumors, while the loss of CD86 expression resulted in a decrease in tumor-infiltrating T lymphocytes in diffuse B-cell large-cell lymphoma [30–35]. YTHDF1 might negatively regulate STAT1 and further facilitated melanoma development, but the roles of STAT1 and CD86 in the general network were not large enough to mitigate the trend of disease development. These genes together promoted the formation of melanoma, as shown in Fig. 4g. To fully explore the role of YTHDF1 or HNRNPA2B1, we assessed their mutations using the cBioPortal Tool (Fig. 5) and found the two genes were mainly amplified in melanoma. Major mutations occurred in the non-coding domain and affected the post-transcription of related proteins by influencing modifications to the proteins such as phosphorylation (Fig. 5c). In addition, the alterations of m^6^A-related genes influenced the patients’ clinical features such as the tumor size, tumor stage, or treatment response (Table 3), indicating their important roles in the progression of melanoma.

However, this research also had some limitations. Firstly, the heterogeneity among the five studies included in Oncomine portal could not be ignored. The heterogeneity might result from patients’ races, treatment or other factors that could have an influence on the expression levels of patients’ transcript files. Secondly, there were few studies including gene qualification documents from both the normal and melanoma sites, therefore the number of samples used to examine the results got from Oncomine was limited. Thirdly, we compared the levels of ten m^6^A regulatory genes between melanoma and the normal groups. Those ten genes included Writers as METTL14, Erasers as FTO, and Readers as YTHDF1, HNRNPA2B1, which were common genes that regulated m^6^A modification in RNA, while with the development new regulatory genes might emerge. Fourthly, the further experiments that explored the mechanism insight into the way m^6^A regulatory genes affected the development of melanoma needed to be performed in the future.

## Conclusion

We systematically examined m^6^A-related genes in melanoma for the first time based on large datasets on publicly available databases. Several sources were used to confirm our results. Statistical analysis revealed the significance of YTHDF1 and HNRNPA2B1 and the combination of the two genes may provide a better approach to diagnose melanoma. Moreover, YTHDF1 or HNRNPA2B1 might interact with RNA processing-related genes such as CDK1/CDK2 and further promote the formation and progression of melanoma. This study may provide new targets for the treatment of melanoma and the evidence to explain the pathophysiology of the disease.

## Supplementary information

**Additional file 1: Table S1.** Characteristics of patients with melanoma on Oncomine. No., number; y, year; n/a, not provided; * one was not provided; T, tumor. **Table S2.** Patient information for Manfred et al. 2018. No., number; NMM, nodular malignant melanoma; SSM, superficial spreading melanoma. **Table S3.** Interactions between the 10 target genes and their parameters. *Q*-value, adjusted *P*-value.

**Additional file 2: Figure S1.** Expression level and ROC curves of ELF3, and YTHDF2. The data was from the GEO dataset online. ROC, receiver operating characteristic curve. *P < 0.05, **P < 0.01. **Figure S2.** Altered characteristics for different genes in melanoma on cBioPortal. (**a**) Alteration frequency in different selected studies. (**b**) The mutation diagram and PTM sites for each gene. PTM, protein post-translational modification.

## Data Availability

The data that support the findings of this study are available from the corresponding author or the first author upon reasonable request.

## Abbreviations

BRAF: B-Raf proto-oncogene
MAPK: Mitogen-activated protein kinase
MEK: Also known as MAP2K7, mitogen-activated protein kinase kinase 7
ERK: Also known as MAPK1, mitogen-activated protein kinase 1
m6A: N6-methyladenosine
METTL3: Methyltransferase like 3
METTL14: Methyltransferase like 14
WTAP: WT1 associated protein
FTO: FTO alpha-ketoglutarate dependent dioxygenase
ALKBH5: alkB homolog 5
YTHDF1: YTH N6-methyladenosine RNA binding protein 1
YTHDF2: YTH N6-methyladenosine RNA binding protein 2
ELF3: E74 like ETS transcription factor 3
HNRNPC: Heterogeneous nuclear ribonucleoprotein C
HNRNPA2B1: Heterogeneous nuclear ribonucleoprotein A2/B1
AML: Acute myeloid leukemia
CSCC: Cervical squamous cell carcinoma
GEO: Gene expression omnibus
ROC: Receiver operating characteristic curve
MCC: Maximal clique centrality
MNC: Maximum neighborhood component
EPC: Edge percolated component
TCGA: The cancer genome atlas
CI: Confidence interval
MW: Mann–Whitney test
Co-gene: The genes included in the correlation analysis
FC: Fold change
FDR: False discovery rate
AUC: Area under curve
PTM: Protein post-translational modification
Glyco: Glycosylation
