# Supplementary material for: Increased expression of YTHDF1 and HNRNPA2B1 as potent biomarkers for melanoma: a systematic analysis
Source: Cancer Cell Int. 2020 Jun 15;20:239. doi: 10.1186/s12935-020-01309-5 (PMC7294677; doi:10.1186/s12935-020-01309-5)
Supplement: Supplementary file 1 — Additional file 1: Table S1. Characteristics of patients with melanoma on Oncomine. No., number; y, year; n/a, not provided; * one was not provided; T, tumor. Table S2. Patient information for Manfred et al. 2018. No., number; NMM, nodular malignant melanoma; SSM, superficial spreading melanoma. Table S3. Interactions between the 10 target genes and their parameters. Q-value, adjusted P-value. [file 12935_2020_1309_MOESM1_ESM.docx]

**Tables**

**Table S1. Characteristics of patients with melanoma on Oncomine.** No., number; y, year; n/a, not provided; * one was not provided; T, tumor.

| **No.** | **Haqq et al., 2005** | | **Talantov et al., 2005** | | **TCGA, 2013** | **Riker et al., 2008** | | **Critchley et al., 2006** |
| --- | --- | --- | --- | --- | --- | --- | --- | --- |
|  | normal | melanoma | normal | melanoma | melanoma | melanoma |  | melanoma |
| Age (Years) |  |  |  |  |  |  |  |  |
| ≤ 60 | 3 | 6 | n/a | 65.51 y (mean) | 258 | - | - | 59.8 y (mean) |
| 60-75 | - | 8 | - | - | 147 | - | - | - |
| ≥ 75 | - | 6 | - | - | 68 | - | - | - |
| Sex |  |  |  |  |  |  |  |  |
| Female | 1 | 4 | 6 | 22 | 183 | - | - | - |
| Male | 2 | 16 | 1 | 23 | 297 | - | - | - |
| Biopsy location |  |  |  |  |  | Metastases |  |  |
| Arm | - | 3 | - | - | - | Lymph node | 22 | - |
| Back | - | 5 | - | - | - | Organ (adrenal and brain) | 16 | - |
| Head and neck | - | - | - | - | - | primary | 42 | - |
| Hand | - | 2 | - | - | - | - | - | - |
| Upper limb | - | - | - | 17 | - | - | - | - |
| Lower limb | - | - | - | 6 | - | - | - | - |
| Face | - | - | - | 5 | - |  |  |  |
| Trunk | - | - | 6 | 9 | 173 | - | - | - |
| Extremities | - | - | - | - | 202 | - | - | - |
| Other | - | 10 | - | 4 | 105 | - | - | - |
| Skin | 3 | - | 1 | 4 | - | - | - | - |
| Thickness (mm) |  |  |  |  |  | Primary thickness (mm) |  |  |
| ≤ 2 | - | 5 | - | - | - | In situ | 2 | - |
| 2-4 | - | 5* | - | - | - | ≤ 1 | 2 | - |
| ≥ 4 | - | 9 | - | - | - | 1-4 | 3 | - |
| T stage |  |  |  |  |  | ≥ 4 | 9 | - |
| T1 | - | - | - | 11 | 42 | - | - |  |
| T2 | - | - | - | 14 | 79 | - | - | - |
| T3 | - | - | - | 16 | 94 | - | - | - |
| T4 | - | - | - | 4 | 156 |  |  | - |
| Other |  |  |  |  | 86 | - | - | - |

**Table S2. Patient information for Manfred et al., 2018.** No., number; NMM, nodular malignant melanoma; SSM, superficial spreading melanoma.

| **Features** | **Classification** | **Nevi (No.)** | **Melanoma (No.)** |
| --- | --- | --- | --- |
| Total | Samples | 23 | 57 |
| Type of Nevus | Dermal | 12 | - |
|  | Compound | 10 | - |
| Type of melanoma | NMM | - | 19 |
|  | SSM | - | 20 |
| Patient age (Years) | ≤ 60 | - | 14 |
|  | 60-75 | - | 22 |
|  | ≥ 75 | - | 21 |
| Breslow  Thickness (mm) | ≤ 2 | - | 22 |
|  | 2-4 | - | 21 |
|  | ≥ 4 | - | 13 |

**Table S3. Interactions between the 10 target genes and their parameters.** *Q*-value, adjusted *P*-value.

| **Gene1** | **Gene2** | ***P*-Value** | ***Q*-Value** | **Tendency** |
| --- | --- | --- | --- | --- |
| HNRNPA2B1 | YTHDF1 | 0.001 | 0.013 | Co-occurrence |
| METTL14 | WTAP | 0.005 | 0.058 | Co-occurrence |
| METTL3 | ALKBH5 | 0.007 | 0.066 | Co-occurrence |
| FTO | YTHDF1 | 0.011 | 0.082 | Co-occurrence |
| FTO | HNRNPA2B1 | 0.028 | 0.141 | Co-occurrence |
| METTL14 | ALKBH5 | 0.03 | 0.141 | Co-occurrence |
| FTO | ALKBH5 | 0.03 | 0.141 | Co-occurrence |
| HNRNPA2B1 | ELF3 | 0.031 | 0.141 | Co-occurrence |
| METTL14 | FTO | 0.035 | 0.145 | Co-occurrence |
| FTO | ELF3 | 0.044 | 0.163 | Co-occurrence |
| ALKBH5 | YTHDF1 | 0.05 | 0.174 | Co-occurrence |
