# Supplementary material for: Increased expression of YTHDF1 and HNRNPA2B1 as potent biomarkers for melanoma: a systematic analysis
Source: Cancer Cell Int. 2020 Jun 15;20:239. doi: 10.1186/s12935-020-01309-5 (PMC7294677; doi:10.1186/s12935-020-01309-5)
Supplement: Supplementary file 2 — Additional file 2: Figure S1. Expression level and ROC curves of ELF3, and YTHDF2. The data was from the GEO dataset online. ROC, receiver operating characteristic curve. *P < 0.05, **P < 0.01. Figure S2. Altered characteristics for different genes in melanoma on cBioPortal. (a) Alteration frequency in different selected studies. (b) The mutation diagram and PTM sites for each gene. PTM, protein post-translational modification. [file 12935_2020_1309_MOESM2_ESM.docx]

**Figures**


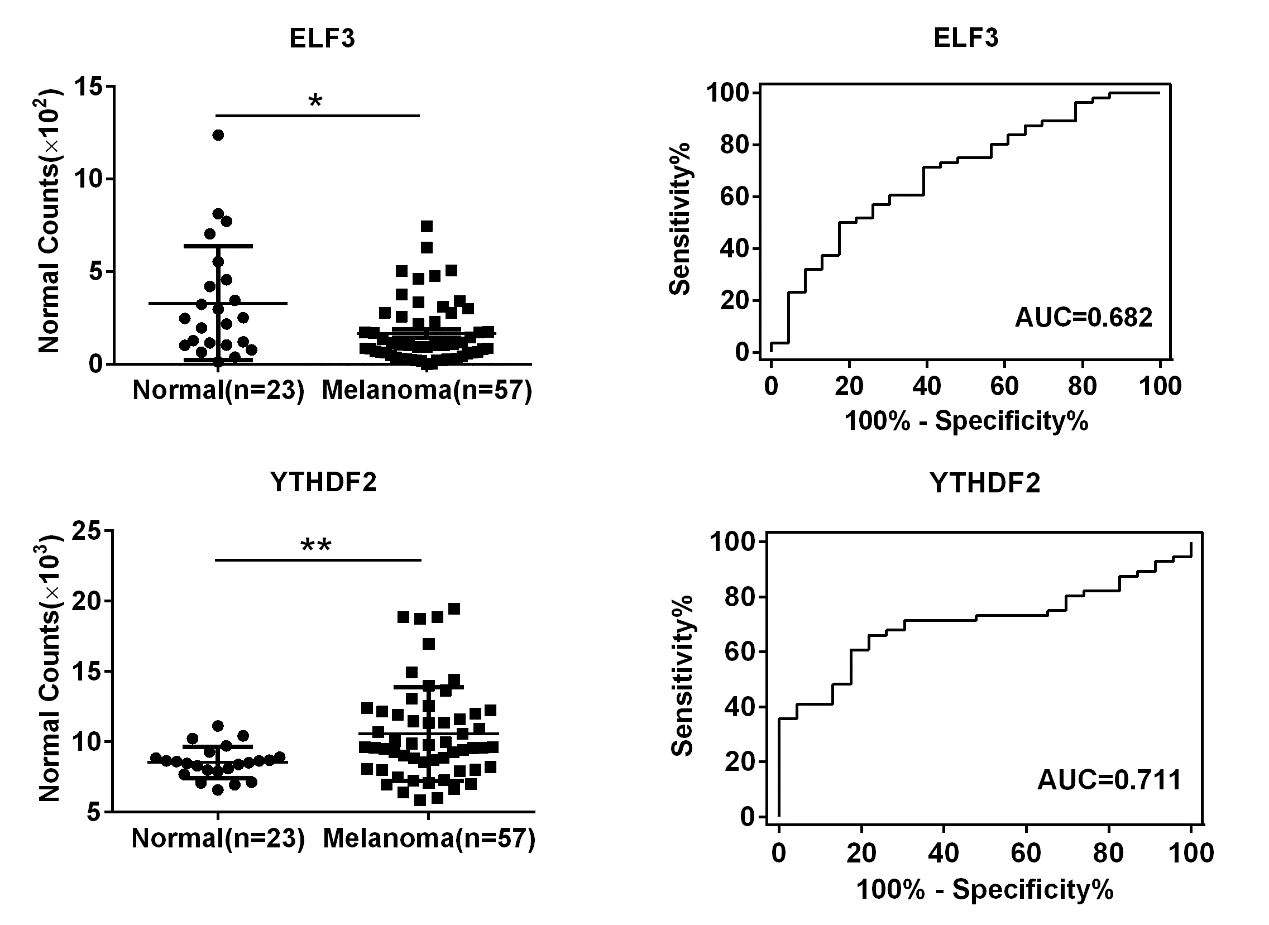


**Figure S1. Expression level and ROC curves of ELF3, and YTHDF2.** The data was from the GEO dataset online. ROC, receiver operating characteristic curve. **P*<0.05, ***P*<0.01.

a


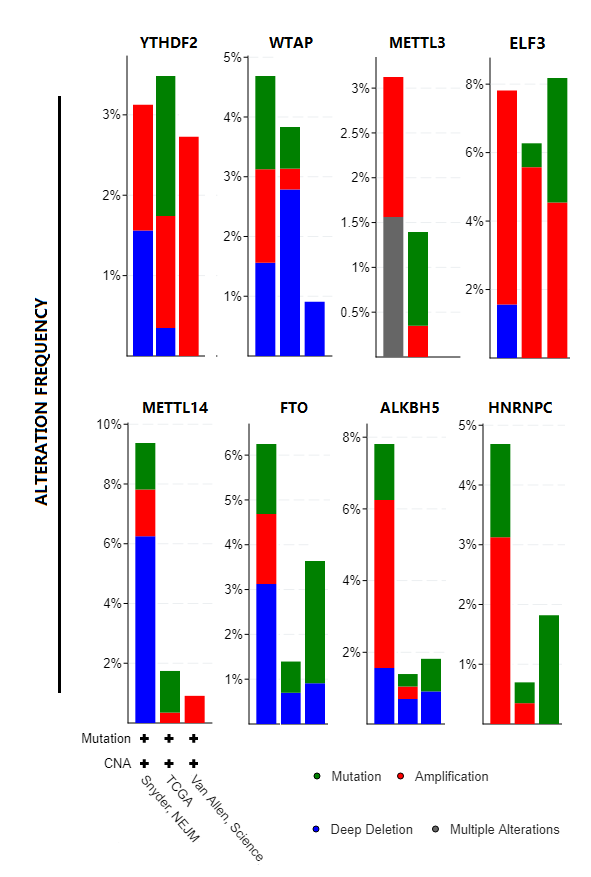


b


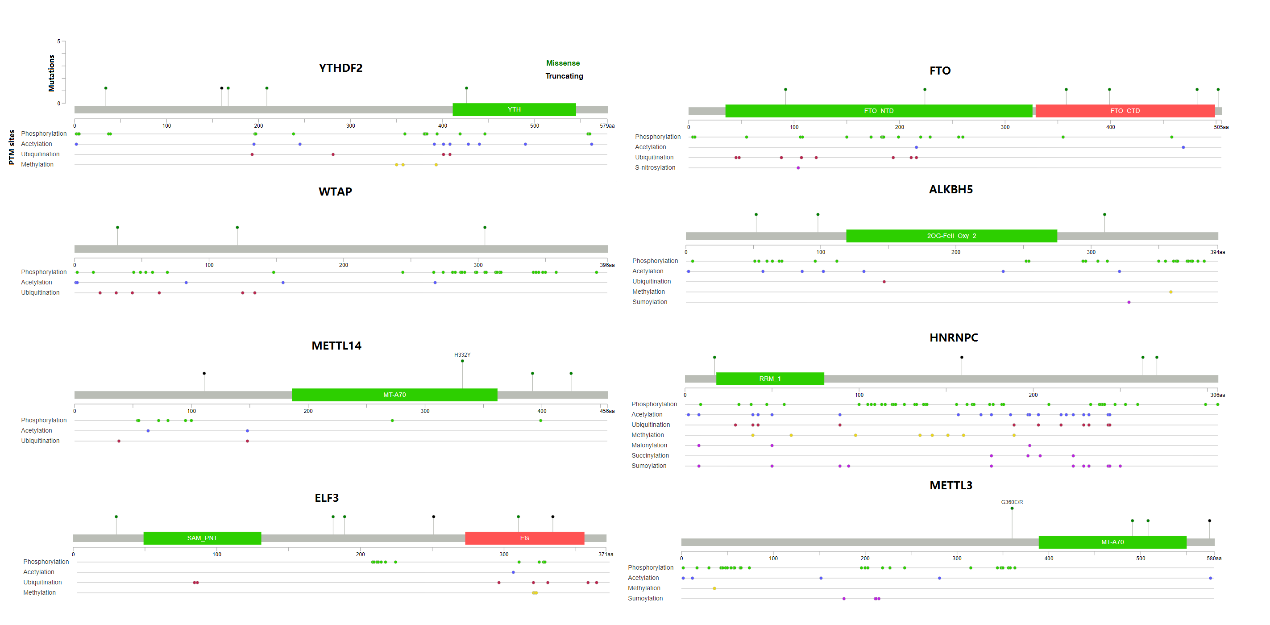


**Figure S2. Altered characteristics for different genes in melanoma on cBioPortal.** (**a**) Alteration frequency in different selected studies. (**b**) The mutation diagram and PTM sites for each gene. PTM, protein post-translational modification.
